# 2-Decenoic Acid Ethyl Ester, a Compound That Elicits Neurotrophin-like Intracellular Signals, Facilitating Functional Recovery from Cerebral Infarction in Mice

**DOI:** 10.3390/ijms13044968

**Published:** 2012-04-19

**Authors:** Yoshitaka Tanaka, Hidefumi Fukumitsu, Hitomi Soumiya, Shinichi Yoshimura, Toru Iwama, Shoei Furukawa

**Affiliations:** 1 Laboratory of Molecular Biology, Gifu Pharmaceutical University, Daigaku-nishi, 1-25-4, Gifu 501-1190, Japan; E-Mails: tanakay@gifu-u.ac.jp (Y.T.); hfukumi@gifu-pu.ac.jp (H.F.): somiya@gifu-pu.ac.jp (H.S.); 2 Department of Neurosurgery, Gifu University Graduate School of Medicine, Yanagido 1-1, Gifu 501-1194, Japan; E-Mails: s-yoshi@gifu-u.ac.jp (S.Y.); tiwama@gifu-u.ac.jp (T.I.)

**Keywords:** 2-decenoic acid ethyl ester, neurotrophin, extracellular signal-regulated kinases 1 and 2, cerebral infarction

## Abstract

In our previous study, we found that trans-2-decenoic acid ethyl ester (DAEE), a derivative of a medium-chain fatty acid, elicits neurotrophin-like signals including the activation of extracellular signal-regulated kinases 1 and 2 (ERK1/2) in cultured mouse cortical neurons. Here, we examined the efficacy of intraperitoneal administration of DAEE on the treatment of a mouse model of the cerebral infarction caused by unilateral permanent middle cerebral artery occlusion (PMCAO). DAEE-treatment (100 μg/kg body weight injected at 0.5, 24, 48, 72 h after PMCAO) significantly restored the mice from PMCAO-induced neurological deficits including motor paralysis when evaluated 48, 72, and 96 h after the PMCAO. Furthermore, DAEE facilitated the phosphorylation of ERK1/2 on the infarction side of the brain when analyzed by Western immunoblot analysis, and it enhanced the number of phosphorylated ERK1/2-positive cells in the border areas between the infarction and non-infarction regions of the cerebral cortex, as estimated immunohistochemically. As the infarct volume remained unchanged after DAEE-treatment, it is more likely that DAEE improved the neurological condition through enhanced neuronal functions of the remaining neurons in the damaged areas rather than by maintaining neuronal survival. These results suggest that DAEE has a neuro-protective effect on cerebral infarction.

## 1. Introduction

**Figure 1:**
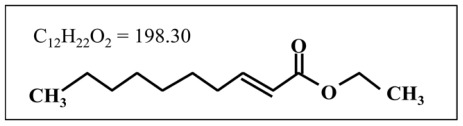
The chemical structure of trans-2-decenoic acid ethyl ester (DAEE).

Previously, we demonstrated that medium-chain fatty acids (MCFAs) with 8–12 carbons and their esters facilitated activation (phosphorylation) of extracellular signal-regulated kinases 1 and 2 (ERK1/2) in cultured embryonic cortical neurons [1,2]. In particular, trans-2-decenoic acid ethyl ester (DAEE: C_12_H_22_O_2_ = 198.30: Figure 1) has the most potent activity among these compounds, and causes the activation of intracellular signal molecules other than ERK1/2 such as Akt and cAMP-responsive element binding protein (CREB), a transcription factor, suggesting that DAEE has neurotrophin-like activities on neurons and thus may be a promising therapeutic tool for certain neurological disorders including cerebral infarction.

Brain-derived neurotrophic factor (BDNF) is a member of the neurotrophin family, and it stimulates neuronal differentiation and maintains survival of various neuronal populations. Recent investigations revealed a relationship between BDNF and various neurological disorders such as Parkinson’s disease [3], Alzheimer’s disease [4], depression [5], and anxiety-related personal trait [6]. These findings suggest the availability of BDNF as a therapeutic tool for particular neurological disorders, and BDNF has been successfully tested in animal models of various diseases. However, clinical trials have been less successful at demonstrating therapeutic efficacy [7]. Many technical and pharmacological issues, for instance, instability of the protein and/or a lack of an appropriate delivery system, remain unsolved. To overcome these problems, the development of stable and small molecules with BDNF-like activity that pass through the blood-brain barrier may represent an alternative approach.

Stroke is the third commonest cause of death in industrialized countries, leading disability in adult and elderly individuals [8]. Cerebral infarction is caused by occlusion of the cerebral arteries [9], and vascular reperfusion therapy is the most effective tool for the prevention of brain injury and reduction of mortality in patients with acute cerebral infarction. In addition to the reperfusion therapy, establishment of effective neuroprotective therapies have been anticipated. For instance, edaravone, a strong free radical scavenger, is considered to have a preventive effect against cerebral infarction [10]. However, the major functional mechanism of edaravone is based on reducing the secondary damage caused by free radicals generated after ischemia. A novel therapeutic tool that can act on neurons directly and preserve their function would contribute to the improvement of the prognosis of patients with cerebral infarction. DAEE may be one such candidate, because it is a compound that can generate neurotrophin-like signals. Therefore, we examined the ameliorative or therapeutic effects of DAEE on a mouse model of the cerebral infarction caused by unilateral permanent middle cerebral artery occlusion (PMCAO).

## 2. Results

### 2.1. Evaluation of Functional Recovery after PMCAO

Recovery of the locomotor function of the right side of body was evaluated by using the scale described later.

**Figure 2:**
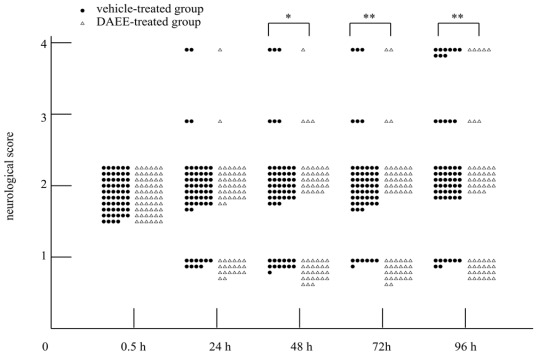
Improvement of neurological conditions by the treatment with DAEE. Values were assessed at 24, 48, 72, and 96 h after permanent middle cerebral artery occlusion (PMCAO). Significance: * *P* < 0.05, ** *P* < 0.01 *vs*. vehicle with Mann-Whitney U test (*n* = 58 [DAEE group] or 60 [vehicle control]). No significant difference was observed in mortality (score = 4) by use of Chi-square test.

PMCAO resulted in moderate motor paralysis of the contralateral side (scale: 2) 0.5 h after the occlusion. Recovery from the motor paralysis was significantly enhanced by intraperitoneal administration of DAEE compared with the paralysis seen in the vehicle-treated group, when evaluated 48, 72, and 96 h after PMCAO. The neurological condition was thus improved in the DAEE-treated group. That is, improvement of the locomotor function from scale 2 to scale 1 occurred earlier in the DAEE-treated group than in the vehicle-treated group (Figure 2). The survival rate of 96 h after PMCAO was higher in the DAEE-treated group than that of the vehicle-treated group (91.7% *vs.* 84.5%, respectively), however, no significant difference was observed (*P* = 0.23, Chi-square test).

### 2.2. Measurement of Infarct Volume

**Figure 3:**
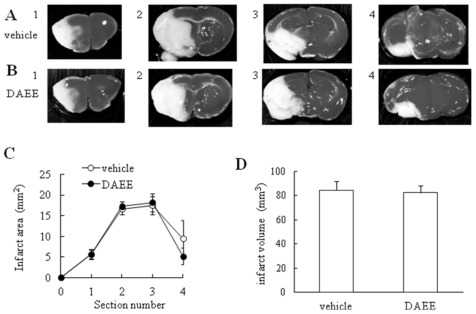
Cerebral infarction after PMCAO in vehicle- or DAEE-treated mice. (**A**) 2,3,5-triphenyltetrazolium chloride (TTC) staining of coronal brain sections (2-mm thick) obtained 96 h after PMCAO from the vehicle-control mice; (**B**) TTC staining of coronal brain sections (2-mm thick) obtained 96 h after PMCAO from the DAEE–treated mice; (**C**) Brain infarct area measured 96 h after PMCAO. Brains were removed, and the forebrains were sliced into five coronal 2-mm sections. The section numbers correspond to the numbers of “A” and “B”. The brain infarct area was not significantly different between the two groups (*vs.* vehicle; Student’s *t*-test; *n* = 6 or 5). The values are expressed as the means ± SEs; (**D**) Brain infarct volume measured 96 h after PMCAO. There was no significant difference in infarct volume between both groups (Student’s *t*-test; *n* = 6 or 5). The values are expressed as the means ± SEs.

Next we examined the infarct volume by staining with 2,3,5-triphenyltetrazolium chloride (TTC; Sigma-Aldrich, Saint Louis, MO, USA) to assess the effect of DAEE treatment quantitatively 96 h after PMCAO. TTC reacts with intact mitochondrial respiratory enzymes to generate a bright-red color that stand in contrast with the pale color of an infarction [11,12]. As the borderlines between normal and infarct areas cannot be detected in microscopic view by TTC staining, Nissl staining is applicable to designate the ischemic region histochemically [13]. The infarction range detected by TTC is perceived to be the same as that detected by Nissl staining. So, we confirmed that the borderlines determined by Nissl staining almost corresponded to those by TTC staining at the microscopic level. Although neuronal death had occurred in the infarct area, no significant differences in the infarct area or volume were found between vehicle- and DAEE-treated groups (Figure 3).

### 2.3. Activation of ERK1/2

**Figure 4:**
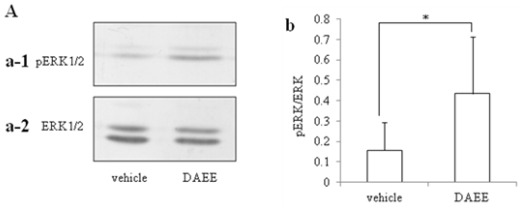

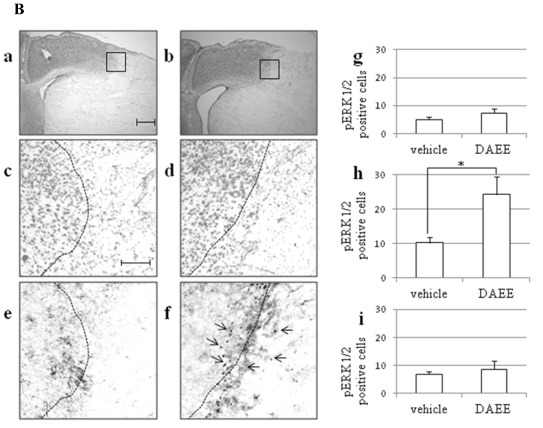
Level and distribution of pERK1/2 in the infracted brain after vehicle- or DAEE-treatment. (**A**) Immunoblot profiles of pERK1/2 (**a-1**) and ERK1/2 (**a-2**) of the vehicle- and DAEE-treated infarct brain 96 h after PMCAO. **b**: Quantitative analysis of the ratio of the intensity of the band of pERK1/2 to that of ERK1/2. The values are expressed as the means ± SEs (*n* = 6). Significance: * *P* < 0.05 *vs.* vehicle (Student’s *t*-test). (**B**) Photographs “**a**” to “**d**” showed Nissl stained profiles, and “**e**” and “**f**” are immunostained ones by anti-pERK1/2. Photographs “**a**” and “**b**” are Nissl stained profiles taken with low-power-magnification. The boxes in photograph “**a**” and “**b**” include the border region between infarct and non-infarct areas, and enlarged as shown in photographs “**c**” and “**d**”. Photographs “**a**” and “**c**” are profiles obtained from the vehicle-treated group and “**b**” and “**d**”, from the DAEE-treated group. The scale bar for both photographs is 500 μm. The dotted lines in photographs “**c**” and “**d**” indicate the borderlines between the infarction and non-infarction areas that were determined in terms of the Nissl-positive cell density. Photographs “**e**” and “**f**” are profiles taken from the adjacent sections immunostained for pERK1/2 in the vehicle- and DAEE-treated groups, respectively. The same dotted lines shown in “**c**” and “**d**” were drawn on the photographs “**e**” and “**f**”. The scale bar indicated in “**c**” is 100 μm and also applicable to “**d**”–“**f**”. The pERK1/2-positive cells were counted in the 3 regions, *i.e.*, the infarction area lateral to the borderline (photograph “**g**”), the ischemic penumbra on the borderline (photograph “**h**”), and the normal area medial to the borderline (photograph “**i**”). Each count was expressed as the number of pERK1/2-positive cells/(200 × 200) μm^2^. These cells, indicated by the arrows in photograph “**f**”, were significantly greater in number in the ischemic penumbra at the borderline (* *P* < 0.05 *vs.* vehicle; Student’s *t*-test; *n* = 3) in the DAEE-treated group than in the vehicle-treated group. However, in the other 2 areas (normal and ischemic), there were no significant differences between the 2 groups (*vs.* vehicle; Student’s *t*-test; *n* = 3).

Activation of ERK1/2 by PMCAO was assessed by Western blot analysis with specific antibody against ERK1/2 or phosphorylated ERK1/2 (pERK1/2). DAEE-treatment significantly raised the level of pERK1/2 4 days after PMCAO to one higher than that of the vehicle-treatment group (Figure 4-A).

### 2.4. Expression of pERK1/2 in Ischemic Penumbra

Nissl staining was performed to determine the border between the normal and infarct areas. The Nissl-positive cells were defined as the cells with a diameter of 10 μm or more. We regarded the normal area as that where the density of Nissl-positive cells was over 5 cells/50 × 50 μm^2^; and the infarct area, as the region with a lower cell density. In this way, we could get polygonal lines. So, we drew curves through the midpoints of each straight line of them fitted by eye. Therefore, we defined the curve as the borderline between the normal and infarct areas. On each consecutive slice, the number of pERK1/2-positive cells was counted in the normal, infarct, and border areas. We counted the numbers in a square area 200 μm on a side with a focus on the borderline as the border area, in the same sized square just medial to the border area as the normal area, and in the square just lateral to it as the infarct area. These areas were counted in high-powered fields on a section through the anterior commissure. Each count was expressed as number of pERK1/2-positive cells/200 × 200 μm^2^ (*n* = 3). In the DAEE-treated group, pERK1/2-positive cells were observed in the border area between the infarct and normal areas, namely, in the area containing Nissl stained neurons, in the cerebral cortex. On the other hand, in the vehicle-treated group, pERK1/2-positive cells in the border area were significantly fewer than those in the DAEE-treated group. The numbers of the pERK1/2-positive cells in the other areas, *i.e.*, normal and infarct areas, were not significantly different between the two groups (Figure 4-B).

### 2.5. Expression of BDNF mRNAs after PMCAO

**Figure 5:**
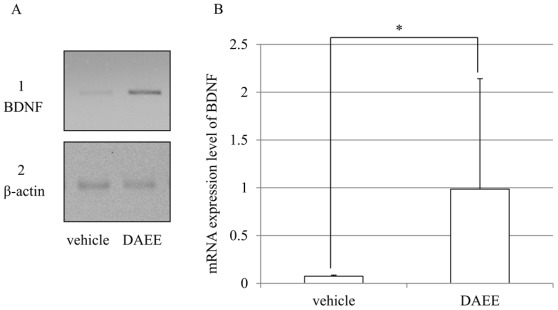
mRNA expression of BDNF in the infracted brain after vehicle- or DAEE-treatment. (**A**) The electrophoresis profiles of BDNF (**A-1**) and β-actin (**A-2**) of the vehicle- and DAEE-treated infarct brain 96 h after PMCAO; (**B**) Quantitative analysis of the ratio of the intensity of the band of BDNF to that of β-actin. The values are expressed as the means ± SEs (*n* = 5–6). Significance: * *P* < 0.05 *vs.* vehicle (Student’s *t*-test).

The mRNA expression of BDNF was investigated by reverse transcription-polymerase chain reaction (RT-PCR). DAEE-treatment significantly raised the expression of BDNF 96 h after PMCAO higher than that of the vehicle-treatment group (Figure 5).

## 3. Discussion

The present results demonstrated the beneficial effects of DAEE on functional recovery after PMCAO, although no significant differences in mortality rate were observed between DAEE and vehicle group 96 h after PMCAO. Furthermore, the size of the infarct area was not significantly different between DAEE- and vehicle- treated groups. It was conspicuous, however, that the level of pERK1/2 in the hemisphere with the infarction was elevated in the DAEE-treated group when judged by Western blotting. The immunohistochemical results confirmed this observation by demonstrating that there were more pERK1/2-positive cells in the DAEE-treated group than in the vehicle-treated group around the borderline (ischemic penumbra) in the cerebral cortex. These results suggest that DAEE preserved the neuronal function in the ischemic penumbra through the activation of ERK1/2 to stimulate expression of neurotrophins and thus contributed to functional recovery. As DAEE did not significantly reduce the infarct size in TTC- and/or Nissl-stained sections, it remains uncertain whether or not DAEE treatment could rescue neurons from dying.

It is well known that ERK1/2 mediates the expression of neuroprotective activity of extracellular neurotrophic factors including neurotrophins. In the nervous system, ERK1/2 is critical for neuronal differentiation and synapse plasticity and may also modulate neuronal survival. Activation of ERK1/2 protects neurons by compensating for the deleterious effects of a damaging insult [14]. The present study showed that pERK1/2-positive cells were localized in the border area between normal and infarct areas in the DAEE-treated group, suggesting that DAEE promoted the activation of ERK1/2 in the neurons damaged by ischemia, perhaps resulting in protection against neuronal death.

In the present study, we found that DAEE did not affect the infarct volume. Jiang *et al.* indicated that BDNF protects neurons against an ischemic insult without affecting the infarct volume estimated by TTC staining [15]. However, they indicated that BDNF prevents the loss of neurons detected by Nissl staining, in the border between normal and infarct areas [15]. Their results suggest that BDNF does not reduce the infarct volume when the gross appearance is observed after TTC staining but that this neurotrophic factor has the ability to prevent neuronal cell loss in the border area when observed microscopically. Therefore, DAEE may also preserve the survival and function of neuronal cells through the activation of ERK1/2 in the ischemic penumbra.

DAEE-treatment enhanced the expression of BDNF mRNAs in present study. These results indicate that DAEE through increased BDNF expression improves the functional recovery after cerebral infarction. Even though DAEE increases the BDNF mRNA (Figure 5), this would not affect the infarction but rather improve the neurological function.

In recent years, evidence has been reported to indicate that omega-3 polyunsaturated fatty acids (PUFAs) such as docosahexaenoic acid (DHA) and eicosapentaenoic acid (EPA) have therapeutic potential in animal models of various neuronal disorders [16–18]. Although limited information is available about the effect of MCFAs on neuronal cells, some recent reports have described such effects. For instance, MCFAs facilitate neurite outgrowth of PC12 cells through the activation of MAPK (p38 mitogen-activated protein kinase)/ERK1/2 pathways [19]; and 10-hydroxy-trans 2-decenoic acid stimulates the neurogenesis of cultured neural stem/progenitor cells, partly mimicking the effect of BDNF [20].

Neurotrophins such as BDNF bind to a specific TrkB receptor tyrosine kinase [21], which causes autophosphorylation of the TrkB receptor to trigger signal transduction cascades including the pathways of MAPK/ERK1/2, phosphatidylinositol 3-kinase (PI3K), and phospholipase C-γ [22]. These signals then pass into the nucleus to activate transcription factors such as CREB, which activation leads to the expression of genes that regulate neuronal functions. The mechanism of DAEE action is unclear at present. However, we have found that DAEE has the ability to stimulate the phosphorylation of epidermal growth factor receptor (EGFR) and of ErbB2 (Abe *et al.*, unpublished results). Although it is unclear at present whether TrkB is involved in the putative action mechanism, it is likely that DAEE stimulated the phosphorylation of ERK1/2 in concentration- and time-dependent manners, and activated Akt located in downstream of PI3K, and CREB through activation of EGFR and/or ErbB2. These observations suggest that DAEE behaves like EGF or vascular endothelial growth factor (VEGF).

Neurotrophic factors including BDNF and EGF have been considered as regulators of neuronal survival and differentiation during development and for maintenance of neuronal functions in adulthood, including synaptic plasticity [23]. Some reports about the beneficial effects of BDNF on cerebral ischemic models were published earlier [24,25]. Furthermore, Ferrer *et al.* reported that BDNF specifically up-regulates its full-length TrkB receptor in cortical neurons in the penumbra area in a rat model of focal ischemia [9]; and Jiang *et al.* indicated that BDNF protects the brain against ischemic insult by modulating local inflammation via regulation of the levels of cellular cytokines and transcription factors in a rat model of PMCAO [15]. However, the clinical trials have been less successful because of many technical and pharmacological issues [26]. These considerations suggest promising availability of DAEE as a therapeutic tool for particular neurological disorders. Furthermore, Hirakwa *et al.* found beneficial effects of DAEE on locomotor function after a spinal cord injury in rats [2].

Nowadays, oxidative stress is considered to be a principal factor promoting brain damage after ischemia. A prime goal of neuroprotective strategy is thus to reduce oxidative damage. In fact, edaravone, a radical scavenger, has been approved as a neuroprotective agent against acute cerebral infarction in Japan [8]. Recent investigations have revealed that propofol, a potent intravenously delivered hypnotic agent, also protects the brain against ischemic injury, by reducing cerebral blood flow, cerebral oxygen metabolism, and intracranial pressure by scavenging free radicals and by decreasing lipid peroxidation [27]. The mechanism underlying the beneficial effects of DAEE on neuronal cells would be different from these mechanisms. In some reports about the effects of edaravone on cerebral infarction, the effective doses of edaravone is 1.5–9 mg/kg body weight in animal models [28–30]. And clinical dose of it is 2.4 mg/kg patients [30]. Compared with these doses of edaravone, the effective dose of DAEE for mice model of cerebral infarction was extremely low (100 μg/kg) in this study. It also indicates the effectiveness of DAEE.

We previously found that the phosphorylation level of ERK1/2 in the cerebral cortex and hippocampus was facilitated in the mice received DAEE intraperitoneally compared with vehicle-treated mice (Makino *et al.*, unpublished data). These results suggest permeability of DAEE and related active conpound through blood-brain barrier. DAEE is a stable molecule with neurotrophic activities, overcoming the various problems of instability and lack of an appropriate delivery system that remain to be problematic with BDNF; and it has novel functional mechanisms of neuroprotection that are different from those of substances and drugs already known. In consideration of these attributes, appending DAEE-treatment to the existing therapy for cerebral infarction may improve the prognosis of the patients.

## 4. Experimental Section

### 4.1. Animal Model of Cerebral Infarction

The experiments were conducted in accordance with the Animal Care Guidance issued by the Animal Experiments Committee of the graduate school of Gifu University and Gifu Pharmaceutical University. All experiments were performed by using male ddY mice (5-week old; body weight 27 to 33 g) purchased from Japan SLC Ltd., (Shizuoka, Japan). Cerebral infarction was produced by permanent occlusion of the left middle cerebral artery. Anesthesia was induced using 2.0 to 3.0% isoflurane and maintained by using 1.5 to 2.0% isoflurane with 70% N_2_O/30% O_2_ delivered by means of an animal general anesthesia machine (Soft Lander, Sin-ei Industry Co Ltd., Saitama, Japan). The regional cerebral blood flow in each mouse was monitored by laser-Doppler flowmetry (Omega-Flow flo-N1; Omegawave Inc., Tokyo, Japan). A flexible probe was fixed to the skull (2 mm posterior and 6 mm lateral to bregma). After a midline skin incision had been made, the left common carotid artery was exposed and occluded. An 8-0 nylon monofilament (Eticon, Somerville, NJ, USA) coated with a mixture of silicone resin (Provil; Hereus Kulzer Inc., Hanau, Germany) was introduced into the left common carotid artery and advanced it along the internal carotid artery until the tip occluded the proximal stem of the middle cerebral artery, as described earlier [31,32].

### 4.2. Administration of the Drug

DAEE was purchased from Sigma-Aldrich Co. LLC. DAEE was dissolved in 0.1% dimethylsulfoxide (DMSO; Wako Pure Chemical Industries Ltd., Osaka, Japan) and diluted with phosphate-buffered saline (PBS). It was administered intraperitoneally into the animals at a dose of 100 μg/kg at 0.5, 24, 48, and 72 h after PMCAO. Control animals received vehicle (PBS) containing 0.1% DMSO without DAEE.

### 4.3. Analysis of Animal Behavior

Mice (vehicle; *n* = 58, DAEE; *n* = 60) were tested for neurological deficits, as described earlier [31] at 0.5, 24, 48, 72, and 96 h after PMCAO. Scoring was done by using the following scale: 0, no observable neurological deficits (normal); 1, failure to extend the right forepaw (mild); 2, circling to the contralateral side (moderate); 3, loss of walking or righting reflex (severe); 4, dead. The investigators were masked as to the group to which each mouse belonged.

### 4.4. Measurement of Infarct Volume

The brains were removed immediately after the mice had been given an overdose of diethyl ether, at 96 h after PMCAO, and cut into 5 serial 2-mm-thick coronal block slices. These slices were immersed for 20 min in a 2% solution of TTC. Image J software was used to measure the unstained areas of the total infarctions, and the infarction volume was calculated as described previously [31].

### 4.5. Western Immunoblot Analysis

The brains were dissected out as described in the previous section. The left hemisphere of the anterior 4th slice was used for Western blotting. Tissues were homogenized in lysis buffer (20 mM Tris-HCl, pH 7.4, containing 150 mM NaCl, 2 mM EDTA, 1% NP-40, 10 μg/mL aprotinin, 10 μg/mL leupeptin, 50 mM NaVO4, 1 mM phenylmethylsulfonyl fluoride [PMSF], 0.1% sodium dodecyl sulfate [SDS] and 1% Na deoxycholate) and then centrifuged, after which the supernatant fluids were collected. Protein concentrations of the supernatants were determined with a BCA Protein Assay Kit (Thermo Scientific, Rockford, IL, USA). Each sample, 2.8 μg protein for ERK1/2 and 7.0 μg protein for pERK1/2 was subjected to 10% SDS-polyacrylamide gel electrophoresis (PAGE) and transferred to polyvinylidene fluoride membranes by electrophoresis. The membranes were blocked for 1 h at room temperature with 5% skim milk (Morinaga Milk Products, Tokyo, Japan) in Tris-buffered saline containing 0.1% Tween 20 (TBS/Tween 20) and then incubated with primary antibody against ERK1/2 or pERK1/2 (1:1000, Cell Signaling Technology, Beverly, MA, USA) for 2 h. After having been washed with TBS/Tween 20, the membranes were incubated with alkaline phosphatase-conjugated anti-rabbit IgG secondary antibody (1:5000 Promega, Madison, WI, USA) for 3 h. Finally the specific protein bands were developed with nitro blue tetrazolium and 5-bromo-4-chloro-3-indrylphosphate *p*-toluidine salt. The intensity of immunoreactive bands was measured by use of image-analysis software (NIH Image J).

### 4.6. Histopathological and Immunohistchemical Study

Brains were removed after cardio-perfusion with 4% (w/v) paraformaldehyde solution prepared in 0.1 M phosphate buffer, pH 7.4 (fixative), 96 h after PMCAO, postfixed in the same fixative overnight, soaked in PBS containing 20% (w/v) sucrose, and frozen in embedding compound (Sakura Finetek, Tokyo, Japan). Coronal sections of 30-μm thickness were prepared with a cryostat (model CM 1800; Leica, Nussloch, Germany), attached to adhesive-coated slides (Matsunami, Osaka, Japan), dried, and soaked in the above fixative for 30 min.

The sections were stained for Nissl substance with 0.1% thionin, dehydrated by passage through an ascending series of ethanol, cleared in xylene, and coverslipped with Euikit (O. Kindler, Freiburg, Germany) [33]. For immunohistochemistry with the chromogen diaminobenzidine (DAB), the sections were treated with TBS/0.3% Triton X-100 containing 0.3% H_2_O_2_ for 3 h, rinsed for 1 h in the same solution, washed 3 times with TBS and blocked for 30 min with 2% (w/v) Block Ace (Dainippon Sumitomo Pharma Co. Ltd., Osaka, Japan) dissolved in TBS (blocking solution). Next, the sections were incubated overnight at 4 °C with antibody against pERK1/2 (1:1000; Chemicon, Temecula, CA, USA). After a wash with TBS, the sections were incubated for 3 h with anti-rabbit IgG goat antibody (1:1000; Vector Laboratories, Burlingame, CA, USA). The sections were then incubated in ABC solution (Elite ABC, Vector Laboratories) with gentle shaking at 4 °C overnight and further treated according to the glucose oxidase-DAB-nickel method. Finally the sections were stained with 3,3′-diaminobenzidine tetrahydrochloride (Dojindo Laboratories, Kumamoto, Japan) for 5–10 min.

### 4.7. RT-PCR

The brains were dissected out as described in the previous section. The left hemisphere of the anterior 4th slice was used for RT-PCR experiment. RNA was prepared from the collected tissues by using TRIZOL (Invirtogen, Carlsbad, CA, USA) according to the manufacture’s instructuins. RT-PCR was performed with PowerScript^TM^ Reverse Transcriptase (Clontech, CA, USA) by following the manufacturer’s instructions. The synthesized cDNAs were amplified with each pair of primers by PCR. The following primer sets were used: β-actin, 5′-GTGGGCCGCTCTAGGCACCAA-3′ and 5′-CTCTTTGATATCACGCACGAT-3′ BDNF, 5′-GGAATTCGAGTGATGACCATCCTTTTCCTTAC-3′ and 5′-CGGATCCCTATCTTCCCCTTTTAATGGTCAGTG-3′. The amplification was carried out under the following conditions: denaturation, 94 °C for 45 s; annealing, 63 °C (for β-actin) or 65 °C (for BDNF) for 45 s; and extension, 72 °C for 45 s. The cycle was repeated 26 times for β-actin, 33 times for BDNF. β-actin was used as an internal control. PCR products were electrophoresed in 2% agarose gels and visualized by Ethidium Bromide staining. The intensity of the bands was analyzed by used of image-analysis software (NIH Image J).

### 4.8. Statistical Analysis

Statistical comparisons were made using two-way analysis of variance followed by Mann-Whitney *U* test, Chi-square test, or Student’s *t*-test.

## 5. Conclusions

We presently found that DAEE acted to facilitate functional recovery of locomotion activity after PMCAO. These findings suggest the possibility that DAEE will become a promising tool for cerebral infarction.
